# Choosing a sensible contrast makes “prevalence bias” irrelevant in screening colonoscopy trials

**DOI:** 10.1007/s10654-025-01301-1

**Published:** 2025-12-03

**Authors:** Marco Piccininni, Vanessa Didelez, Mats J. Stensrud

**Affiliations:** 1 Institute of Mathematics, École Polytechnique Fédérale de Lausanne, Lausanne, Switzerland; 2 Leibniz Institute for Prevention Research and Epidemiology - BIPS, Bremen, Germany; 3 Department of Mathematics and Computer Science, University of Bremen, Bremen, Germany

**Keywords:** Screening colonoscopy, Prevalence bias, Risk ratio, Effect heterogeneity

## Abstract

Screening colonoscopy has been shown to reduce colorectal cancer incidence. However, the magnitude of this effect is debated. There is concern that some trial participants already had colorectal cancer at baseline. The screening procedure could not prevent disease occurrence in these participants, leading to “prevalence bias”. Some authors have argued that the effect of interest is confined to participants without disease at baseline, and failing to exclude prevalent cases supposedly leads to effect underestimation. Yet, the issue is debated, with other authors arguing that conventional randomized trials provide the effects that are most relevant to public health. Here we present new, formal arguments that clarify misconceptions in this debate. We show that, under mild assumptions, the so-called “prevalence bias” is not a concern when researchers are interested in estimating risk differences, rather than risk ratios. This is because of a statistical property of the causal risk difference when outcomes are rare, called “doomed-selection stability”.

**Supplementary Information:**

The online version contains supplementary material available at 10.1007/s10654-025-01301-1.

## Introduction

The Nordic-European Initiative on Colorectal Cancer (NordICC) trial, has shown that invitation to screening colonoscopy reduces colorectal cancer incidence at 10 years [1]. The reduction is likely due to the removal of benign precursors (polyps), which is an integral part of the screening procedure. Nevertheless, the magnitude of this effect is debated. One issue is that some trials participants already have colorectal cancer, or pre-clinical manifestations of it, at baseline. The screening procedure cannot reasonably prevent the disease from occurring in these prevalent cases. This issue sparked a broader methodological debate about causal estimands and trial designs.

Some authors have argued that the presence of prevalent cases of colorectal cancer at baseline complicates the interpretation of the randomized controlled trials (RCTs) [2–7]. For example, Brenner et al. consider the exclusion of prevalent cases an “important prerequisite” of a properly conducted RCT [2], and their inclusion a violation of “a key principle of prevention trials” [3]. It has been argued that the contrast of interest is the effect among the subset of participants that are free of the disease at baseline [2]. Since published RCTs also include prevalent cases, it has been argued that these RCT underestimate the effect of colorectal cancer screening [2, 3, 6]. This problem was named “prevalence bias” [2, 3, 8].

Some researchers have acknowledged the issue of prevalent cases, but nevertheless they believe these cases should be included in the analysis for practical purposes [6, 9]. Others have argued that the public health relevant effect is the one in the entire population, including the prevalent cases [10]. These points are central in the discussion of benefits and cost-effectiveness of colorectal cancer screening programs [4, 7, 9].

Here we present new, formal arguments that clarify misconceptions in this debate: we show that under mild assumptions the so-called “prevalence bias” is not a concern when researchers are interested in estimating risk differences (rather than risk ratios). This is because of a mathematical property of the causal risk difference when outcomes are rare, called “doomed-selection stability” [11].

## The NordICC trial’s “prevalence bias” controversy

The Nordic-European Initiative on Colorectal Cancer (NordICC) was a pragmatic RCT involving presumptively healthy individuals between 55 and 64 drawn from population-based registries in Poland, Norway, Sweden, and the Netherlands conducted between 2009 and 2014 [1]. Participants were randomized 1:2 to be invited to a screening colonoscopy or not [1]. The published results included 84,585 participants from Poland, Norway and Sweden and showed a lower (cumulative) risk of colorectal cancer at 10 years in the invited group compared to the usual-care group [1]. NordICC represented the first RCT for long-term effects of screening colonoscopy, and the estimated risk ratio for colorectal cancer at 10 years was 0.82, a smaller effect compared to previously published observational studies [2, 4, 7, 9, 12].

Brenner and colleagues argued that the NordICC trial was affected by “prevalence bias” [2, 3, 8], and this, at least partially, explained the risk ratio closer to 1 compared to previous estimates [2, 4, 5, 7]. In 2023, Brenner and colleagues provided calculations approximating the NordICC risk ratio, had all incident colorectal cancer been prevented by the screening, to be 0.70 [5]. In the same year, Brenner et al. published an essay approximating the risk ratio of the NordICC trial had prevalent cases been excluded, under different hypothetical prevalences of colorectal cancer at baseline [2]. They suggested that the risk ratio could vary substantially and concluded that “non-preventable CRC cases diminished reported screening effects” in the NordICC trial [2].

An author of the original NordICC publication agreed that prevalent cases should, in theory, have been excluded from the RCT: Song and Bretthauer write “For any epidemiologic study interested in disease incidence, individuals who have already developed the disease at baseline should be excluded from the study because they already have been diagnosed and thus cannot contribute meaningfully to analyses” [9]. However, the exclusion of prevalent cases is deemed unfeasible in practice by Song and Bretthauer [9]; in their words, the impossibility of counting prevalent cases is a concern because “there are no reliable statistical analyses which can tease out the true screening benefits without counting them” [9]. The debate highlights the need for methodological developments to tackle the issue of “prevalence bias” [6, 8, 9].

The issue concerns the choice of effect or, more specifically, of the intended target population: are we targeting the population that is generally eligible to attend screening colonoscopies, or only those who are free of colorectal cancer? Next, we will consider a development that helps clarify the debate; the question of the population does not matter if we choose certain contrasts.

### Stability of the risk difference

Consider the data reported by Brenner and colleagues [2]. We will specifically consider their intention-to-screen analysis, and exclude the scenario “Plausible lower bound” because it seems to include some typos (see Table) [2]. The first row of the Table shows the values attributed to the original NordICC trial. The other rows represent hypothetical results after excluding prevalent cases, when the prevalence of colorectal cancer at baseline ranges from “Theoretical minimum” to “Theoretical maximum” [2]. As in the original analyses [2], we report the risk ratio and the cumulative incidences at 10 years in the treatment arm (invited to screen) and the control arm (usual care). We additionally calculate the “survival ratio”, corresponding to the risk ratio for the event “not having colorectal cancer by year 10”, and the risk difference, corresponding to the difference in cumulative cancer incidence between the treatment and control arms.

As argued by Brenner et al., the risk ratio varies dramatically, between 0.48 and 0.82, when hypothetically excluding different assumed proportions of prevalent cases from the analysis [2]. However, the survival ratio and the risk difference are identical in the five different scenarios. Therefore, the risk difference reported in the NordICC trial [1], corresponds to the risk difference estimates when excluding prevalent cases at baseline in all proposed scenarios.

**Table:** 

Scenario name	Percentage of prevalent cases at baseline (%)	10-year incidence, treatment arm (%)	10-year incidence, control arm (%)	Risk Ratio	Survival Ratio	Risk Difference (perc. points)
Reported in NordICC trial	None excluded	0.98	1.20	0.82	1.0022	− 0.22
Theoretical minimum	0.22	0.76	0.98	0.78	1.0022	− 0.22
Base-case scenario	0.52	0.46	0.68	0.68	1.0022	− 0.22
Plausible higher bound	0.58	0.40	0.62	0.65	1.0022	− 0.22
Theoretical maximum	0.78	0.20	0.42	0.48	1.0022	− 0.22

*Table* Results from the analysis of Brenner et al. [2]. The values for the first five columns were extracted from Table 3 of Brenner et al. 2023 [2]. In the first row, the values attributed to the original NordICC trial are reported. The other rows represent hypothetical scenarios in which different prevalences of colorectal cancers at baseline are assumed, and the analyses are reported excluding prevalent cases. We additionally calculated the survival ratio (column six) and the risk difference (column seven) for each scenario using the figures reported by Brenner et al. [2].

### Why are the survival ratio and the risk difference unaffected by “prevalence bias”?

Brenner et al. [2] assumed that the proportion of prevalent cases is the same in the two arms, due to randomization. Additionally, they implicitly assumed that prevalent cases would always be diagnosed with colorectal cancer during the 10-year study period, regardless of the treatment arm they were assigned to. Thus, they assumed that individuals who have colorectal cancer at baseline are “doomed” to be diagnosed during the study period.

Certain contrasts, such as the survival ratio, are stable to doomed-selection [11]. That is, excluding doomed participants from a large RCT without losses to follow-up, does not change the survival ratio [11].

It is a well-known epidemiologic fact that, when the outcome is rare under no treatment, the risk difference can be expressed as a simple transformation of the survival ratio [11, 13]. Thus, when the outcome is rare, the risk difference is approximately stable with respect to doomed-selection [11]. In the scenario considered in the Table, the incidence for colorectal cancer in the control group is below 1.5%, satisfying this rare event condition. Therefore, removing preclinical colorectal cancers from the trial does not meaningfully change the risk difference. This is an important result, as we cannot practically identify all individuals with preclinical colorectal cancer at baseline; our results show that under mild assumptions we do not need to.

The notion that individuals with preclinical colorectal cancer at baseline are “doomed” is a formalization of the statement that “people who already have the disease should be excluded as the intervention can no longer prevent it” [2]. Similarly, it has been claimed that “There is no way screening could have prevented these prevalent cancers, even though it could have led to their earlier detection” [3]. The assumption that all prevalent cases are doomed may be violated if the length of the study is short, if screening leads to overdiagnosis, or if there are competing events (e.g., death due to other causes). Then, a prevalent case is not necessarily diagnosed with cancer in the no screening arm; for example, they might not develop any cancer symptoms during the study period, or they might die without being diagnosed. If prevalent cases are always diagnosed in the treatment arm, but not necessarily in the control arm, then the survival ratio in the entire population will be smaller or equal than the survival ratio calculated after excluding the prevalent cases. However, if we consider sufficiently long follow-up, a population of young adults who have a small risk of dying, and overdiagnoses are unlikely, the doomed assumption is plausible.

In the Supplementary material we give a formal proof of the doomed-selection stability of the survival ratio and of the approximate doomed-selection stability of the risk difference. We also present a simulation study to give numerical support to our theoretical results and discuss the scenario in which prevalent cases are always diagnosed in the treatment arm but not necessarily diagnosed in the control arm.

## Discussion

The debate on prevalent cases in screening colonoscopy RCTs is active [2–10]. Some have considered the inability to exclude prevalent cases as an inherent limitation of RCTs for screening programs [2]. Others recognized the issue of prevalent cases, but still considered RCTs including prevalent cases the preferred way to answer causal questions about screening [9]. There are also authors who have questioned that “prevalence bias” is an issue, arguing that the effect in the whole population is the most practically relevant [10].

In the words of Brenner et al., the ideal (but unfeasible) RCT would exclude prevalent cases: “In theory, in such a setting, the effectiveness of CRC prevention could still be assessed in a randomized design in which participants with findings of prevalent CRC at colonoscopy would be excluded and the remaining participants would be randomized in such a way that precancerous lesions would be removed in the intervention group only but not in the control group. Obviously, such an approach would be unethical and not be a viable option” [2].

Also, researchers who agree that, ideally, prevalent cases should be excluded do not necessarily believe that we can reliably estimate effects restricted to non-prevalent cases. For example, Brenner et al. reanalyzed the NordICC trial to study such effects [2, 4, 7]. However, these reanalyses inevitably rely on strong assumptions. Song and Bretthauer considered the methods employed by Brenner et al. “crude and based on several uncertain assumptions” [6]. They further considered the issue of prevalent cases still open, as counting the prevalent cases in an RCT is unfeasible and “there are no reliable statistical analyses which can tease out the true screening benefits without counting them.” [9].

We showed that, under mild assumptions, the risk difference obtained from an RCT is approximately stable to the inclusion of prevalent cases, given that the outcome is rare. When a researcher is interested in the risk difference, the effect in the whole population adequately approximates the effect in units without disease at baseline. Furthermore, there is no need to “count” the prevalent cases to estimate the risk difference only among non-prevalent cases. In all possible scenarios with different prevalences of colorectal cancer at baseline considered by Brenner et al. [2], the exclusion of prevalent cases gave the same risk difference. While we focused on the intention-to-screen effect in the NordICC trial [1], the same argument holds also for estimates of per-protocol effects from randomized trials [14], or for estimates obtained from observational claims data [15]. This is because, unlike the risk ratio, the survival ratio and the risk difference (when the outcome is rare) are stable to doomed-selection [11].

Our results offer a new perspective on the statement that “the current practice of counting prevalent cases in clinical trials inevitably leads to substantial underestimation of reported screening effects in all theoretically possible and plausible scenarios” [8]. Specifically, the risk difference estimate is not expected to meaningfully change in RCTs for colorectal cancer screening, regardless of the inclusion or exclusion of prevalent cases.

This is an important result, as researchers often consider the risk difference a more relevant contrast when evaluating policy decisions. For example, in the NordICC original publication the authors highlighted the importance of considering absolute risks and effects (instead of relative ones) when planning cancer screening programs [1].

Interestingly, Brenner et al. proposed the presence of prevalent cases at baseline as an explanation of the different risk ratio estimate obtained by the NordICC trial compared to other observational studies [2, 4, 7, 8]. A recent investigation by Braitmaier et al. suggested instead that the difference in effect estimates between the RCT and observational studies is due to non-alignment of “time zero” in the observational studies [10]. Our results motivate a simple falsification strategy for the claim: if the only difference between observational studies and the NordICC trial risk ratio estimates is due to the presence of more prevalent cases, then the risk difference estimates should coincide.

While we considered the effect of screening colonoscopy, our arguments apply more generally to RCTs that study preventive measures to reduce the incidence of a disease. Brenner et al. suggested that prevalent cases can affect the evaluation of other screening programs as well [2, 4]. For example, recently RCT data were reanalyzed to estimate the effect of flexible sigmoidoscopy screening on colorectal cancer incidence excluding prevalent cases [3]. Also in this case, the risk ratio can change substantially when excluding prevalent cases. However, under mild assumptions, the risk difference is expected to remain approximately the same.

## Supplementary Information

Below is the link to the electronic supplementary material.

Supplementary Material 1
