# Supplementary material for: Choosing a sensible contrast makes “prevalence bias” irrelevant in screening colonoscopy trials
Source: Eur J Epidemiol. 2025 Dec 3;40(11):1275–9. doi: 10.1007/s10654-025-01301-1 (PMC12695937; doi:10.1007/s10654-025-01301-1)
Supplement: Supplementary file 1 — Supplementary Material 1 [file 10654_2025_1301_MOESM3_ESM.docx]

**Choosing a sensible contrast makes “prevalence bias” irrelevant**

**in screening colonoscopy trials**

**Supplementary Materials**

Marco Piccininni^1^, Vanessa Didelez^2,3^, Mats J. Stensrud^1^

^1^ Institute of Mathematics, École Polytechnique Fédérale de Lausanne, Lausanne, Switzerland

^2^ Leibniz Institute for Prevention Research and Epidemiology - BIPS, Bremen, Germany

^3^ Department of Mathematics and Computer Science, University of Bremen, Bremen, Germany

Corresponding author:

Mats J. Stensrud

EPFL SB MATH BIOSTAT

Station 8

1015 Lausanne

mats.stensrud@epfl.ch

**Table of Contents**

**Technical Appendix**

**Scenario in which prevalent cases are not necessarily diagnosed under control**

**Simulation**

**Supplementary figures**
Figure S1
Figure S2

**References**

**Technical appendix**

Suppose that we have data on individuals that represent independent draws from a near-infinite superpopulation. Define the binary treatment variable as $A$, where $A=1$ indicates that the unit is invited to be screened (intervention group) and $A=0$ indicates no invitation (control group). Define a binary outcome variable $Y$, indicating whether the individual has obtained ($Y=1$) or not ($Y=0)$ a diagnosis of colorectal cancer by the end of the study period. Let $Y^{a}$ be the potential outcome [1], that is, the outcome had the individual, possibly contrary to facts, received treatment $A=a$. The causal risk difference [1] is defined as

**Definition 1.** $CRD=E\left[ Y^{1} \right]-E\left[ Y^{0} \right]$.

That is, the $CRD$ is the difference between the probability of the outcome under intervention and the probability of the outcome under control.

The causal “survival ratio” [2] is instead defined as

**Definition 2.** $CSR=\frac{1-E\left[ Y^{1} \right]}{1-E\left[ Y^{0} \right]}$.

That is, the $CSR$ is the ratio between the probability of not having the outcome under intervention and the probability of not having the outcome under control.

Define $P$ has a binary variable indicating whether an individual had the disease already at baseline ($P=1$) or not ($P=0)$. The variable $P$ therefore indicates whether the individual is a “prevalent case” (or a “preclinical colorectal cancer”) at baseline.

We assume that there exist some individuals with the disease at baseline, but not all individuals have the disease at baseline.

**Assumption 1.** $0<E[P]<1$.

The contrasts in Definitions 1 and 2 are marginal effects, defined in the entire population. We now formalize the target estimand from Brenner et al. [3]: the (conditional) contrast of expected counterfactuals only in the subgroup of individuals who did not have the disease at baseline. Then, define the causal risk difference and the causal survival ratio for units without the disease at baseline.

**Definition 3.** ${CRD}_{P=0}=E\left[ Y^{1}|P=0 \right]-E\left[ Y^{0}|P=0 \right]$

**Definition 4.** ${CSR}_{P=0}=\frac{1-E\left[ Y^{1}|P=0 \right]}{1-E\left[ Y^{0}|P=0 \right]}.$

The marginal effect can be estimated from a practically and ethically feasible randomized experiment. However, an ideal RCT that identifies the effect in the subset of disease-free individuals, is unfeasible to conduct [3]. This is because it is challenging, although in principle doable, to measure $P$ at the beginning of the trial.

We will assume that the probability of experiencing the outcome under no intervention is low; that is, the outcome is rare. This assumption is plausible when colorectal cancer is the outcome, and we consider a cohort of adults followed for 10 years, as in the NordICC trial [4].

**Assumption 2.** $E\left[ Y^{0} \right]<\varepsilon$, where $\varepsilon$ is a small positive number.

We finally assume that individuals who have the disease at baseline are always diagnosed with colorectal cancer by the end of the study period, regardless of the intervention:

**Assumption 3.** $E\left[ Y^{1}|P=1 \right]=E\left[ Y^{0}|P=1 \right]=1$.

Assumption 3 formalizes the statement from Brenner et al. that “people who already have the disease should be excluded as the intervention can no longer prevent it” [3], indeed the intervention has no effect for this subset of patients.

We emphasize that Assumption 3 was also made implicitly by Brenner et al. when they reanalysed data from the NordICC trial [3]. They assumed that the prevalent cases would have been diagnosed by the end of the study period, regardless of the trial arm they had been assigned to. This assumption is implicit in the subtraction Brenner et al. make to obtain the “prevalence-corrected” risks [3]. A similar assumption was also leveraged in the reanalysis of sigmoidoscopy screening [5].

Under Assumptions 1-3, all the causal contrasts in Definitions 1-4 are well defined, as the conditioning sets are non-empty and the denominators are different from zero.

**Proposition 1.** Under Assumptions 1-3, $CSR={CSR}_{P=0}$ and $CRD\approx{CRD}_{P=0}$.

That is, the marginal causal survival ratio is equal to the causal survival ratio among units without disease at baseline, and the marginal causal risk difference is approximately equal to the causal risk difference among units without disease at baseline. This result is useful because we cannot practically observe whether an individual has $P=0$ at baseline. We present a formal proof below. Briefly, the first equality, $CSR={CSR}_{P=0}$, comes from the stability to doomed-selection of the survival ratio [6]. This equality actually holds even if the outcome under no treatment is not rare (i.e., if Assumption 2 holds only with $\varepsilon=1$) [6]. The approximation $CRD\approx{CRD}_{P=0}$, instead, can be derived from the fact that when the outcome under no treatment is rare, the risk difference approximates one minus the survival ratio [2,6]. While we assumed $E\left[ P \right]>0$, the case where $E\left[ P \right]=0$ is uninteresting because the marginal and conditional effects exactly coincide.

Proposition 1 expresses a relationship between causal parameters in the population. These causal parameters can be trivially identified in a randomized controlled trial. In an ideal randomized trial, the assumptions of consistency ($Y^{a}=Y$ when $A=a$, for every $a$), exchangeability ($Y^{a}⫫A$, for every $a$), and positivity ($0<E[A]<1$) are guaranteed to hold [1]. Under these assumptions, it is possible to identify both the $CSR$ and the $CRD$, since $E\left[ Y^{a} \right]=E[Y|A=a]$ for every $a$ [1].

**Proof of Proposition 1**

We will first prove the first equality in Proposition 1

$$CSR\overset{\left( D2 \right)}{=}\frac{1-E\left[ Y^{1} \right]}{1-E\left[ Y^{0} \right]}=\frac{1-E\left[ Y^{1}|P=1 \right]\cdot E\left[ P \right]-E\left[ Y^{1}|P=0 \right]\cdot\left( 1-E\left[ P \right] \right)}{1-E\left[ Y^{0}|P=1 \right]\cdot E\left[ P \right]-E\left[ Y^{0}|P=0 \right]\cdot\left( 1-E\left[ P \right] \right)}$$

$$\overset{\left( A3 \right)}{=}\frac{1-E\left[ P \right]-E\left[ Y^{1}|P=0 \right]\cdot\left( 1-E\left[ P \right] \right)}{1-E\left[ P \right]-E\left[ Y^{0}|P=0 \right]\cdot\left( 1-E\left[ P \right] \right)}=\frac{\left( 1-E\left[ Y^{1}|P=0 \right] \right)\cdot\left( 1-E\left[ P \right] \right)}{\left( 1-E\left[ Y^{0}|P=0 \right] \right)\cdot\left( 1-E\left[ P \right] \right)}$$

$$=\frac{1-E\left[ Y^{1}|P=0 \right]}{1-E\left[ Y^{0}|P=0 \right]}\overset{\left( D4 \right)}{=}{CSR}_{P=0}.$$

Assumption 1 and 2, ensure that all quantities are well-defined. This proof follows closely the argument in Piccininni and Stensrud for the immune-selection stability of the risk ratio. We emphasize that the proven identity holds even if the outcome under no treatment is not rare (i.e., if Assumption 2 holds only with $\varepsilon=1$) [6].

We can rewrite one minus the marginal causal survival ratio as

$$1-CSR\overset{\left( D2 \right)}{=}1-\frac{1-E\left[ Y^{1} \right]}{1-E\left[ Y^{0} \right]}=\frac{1-E\left[ Y^{0} \right]-1+E\left[ Y^{1} \right]}{1-E\left[ Y^{0} \right]}=\frac{E\left[ Y^{1} \right]-E\left[ Y^{0} \right]}{1-E\left[ Y^{0} \right]}\overset{\left( D1 \right)}{=}\frac{CRD}{1-E\left[ Y^{0} \right]}.$$

And similarly, when conditioning on $P=0$,

$$1-{CSR}_{P=0}\overset{\left( D4 \right)}{=}1-\frac{1-E\left[ Y^{1}|P=0 \right]}{1-E\left[ Y^{0}|P=0 \right]}=\frac{E\left[ Y^{1}|P=0 \right]-E\left[ Y^{0}|P=0 \right]}{1-E\left[ Y^{0}|P=0 \right]}\overset{\left( D3 \right)}{=}\frac{{CRD}_{P=0}}{1-E\left[ Y^{0}|P=0 \right]}.$$

Therefore, from the first equality in Proposition 1 we have

$$\frac{CRD}{1-E\left[ Y^{0} \right]}=1-CSR=1-{CSR}_{P=0}=\frac{{CRD}_{P=0}}{1-E\left[ Y^{0}|P=0 \right]}.$$

This equality holds regardless of how rare the outcome is, as long as the denominators are non-zero.

Thus, $CRD$ and ${CRD}_{P=0}$ will be approximately equal when $1-E\left[ Y^{0} \right]$ and $1-E\left[ Y^{0}|P=0 \right]$ are approximately equal. We will now show that, under Assumption 2, this is the case.

Under Assumption 2, that is when the outcome is rare under no treatment,
$|(1-E\left[ Y^{0} \right])-1|<\varepsilon$. Moreover, since

$$\varepsilon>E\left[ Y^{0} \right]=E\left[ Y^{0}|P=1 \right]\cdot E\left[ P \right]+E\left[ Y^{0}|P=0 \right]\cdot\left( 1-E\left[ P \right] \right)$$

$$\overset{\left( A3 \right)}{=}E\left[ P \right]+E\left[ Y^{0}|P=0 \right]\cdot\left( 1-E\left[ P \right] \right)=E\left[ P \right]+E\left[ Y^{0}|P=0 \right]-E\left[ Y^{0}|P=0 \right]\cdot E\left[ P \right]$$

$$=E\left[ Y^{0}|P=0 \right]+E\left[ P \right](1-E\left[ Y^{0}|P=0 \right])\geq E\left[ Y^{0}|P=0 \right]$$

it also follows that $|(1-E\left[ Y^{0}|P=0 \right])-1|<\varepsilon$.

That is, under Assumption 2, both $1-E\left[ Y^{0} \right]$ and $1-E\left[ Y^{0}|P=0 \right]$ are approximately equal to 1. Therefore, we have shown that

$$CRD\approx1-CSR=1-{CSR}_{P=0}\approx{CRD}_{P=0}$$

which completes the proof.

While we relied on Assumption 2 to justify that $1-E\left[ Y^{0} \right]$ and $1-E\left[ Y^{0}|P=0 \right]$ are approximately equal, other assumptions, such as a low $E\left[ P \right]$ could be alternatively invoked to show the stability of the risk difference. However, we emphasize that Assumption 2 can be falsified in a randomized experiment without measuring $P$.

**Scenario in which prevalent cases are not necessarily diagnosed under control**

As discussed in the main text, Assumption 3 may be violated if the length of the study is short, if there exist competing events (such as death), or if screening leads to overdiagnosis. In these cases, an individual with the disease at baseline might remain undiagnosed without screening. This happens, for example, if the individual dies or never develops symptoms during the study period.

Suppose that prevalent cases (however we decide to define them), are always diagnosed under treatment, but we are agnostic to whether they would be diagnosed under control:

**Assumption 4.** $E\left[ Y^{1}|P=1 \right]=1$.

Then, it is possible to identify a lower bound for the causal survival ratio in the subgroup of non-prevalent cases, without measuring $P$.

**Proposition 2.** Under Assumptions 1, 2, and 4, we have that: $CSR\leq{CSR}_{P=0}$.

Proposition 2 states that when all prevalent cases are diagnosed under treatment, the marginal causal survival ratio is always lower or equal than the causal survival ratio among non-prevalent cases only.

**Proof of Proposition 2**

$$CSR\overset{\left( D2 \right)}{=}\frac{1-E\left[ Y^{1} \right]}{1-E\left[ Y^{0} \right]}=\frac{1-E\left[ Y^{1}|P=1 \right]\cdot E\left[ P \right]-E\left[ Y^{1}|P=0 \right]\cdot\left( 1-E\left[ P \right] \right)}{1-E\left[ Y^{0}|P=1 \right]\cdot E\left[ P \right]-E\left[ Y^{0}|P=0 \right]\cdot\left( 1-E\left[ P \right] \right)}$$

$$\overset{\left( A4 \right)}{=}\frac{1-E\left[ P \right]-E\left[ Y^{1}|P=0 \right]\cdot\left( 1-E\left[ P \right] \right)}{1-E\left[ Y^{0}|P=1 \right]\cdot E\left[ P \right]-E\left[ Y^{0}|P=0 \right]\cdot\left( 1-E\left[ P \right] \right)}$$

$$=\frac{\left( 1-E\left[ Y^{1}|P=0 \right] \right)\cdot\left( 1-E\left[ P \right] \right)}{1-E\left[ Y^{0}|P=1 \right]\cdot E\left[ P \right]-E\left[ Y^{0}|P=0 \right]\cdot\left( 1-E\left[ P \right] \right)}$$

$$=\frac{1-E\left[ Y^{1}|P=0 \right]}{\frac{1-E\left[ Y^{0}|P=1 \right]\cdot E\left[ P \right]}{1-E\left[ P \right]}-E\left[ Y^{0}|P=0 \right]}$$

where Assumption 1 guarantees that the denominators are non-zero.

And since $\frac{1-E\left[ Y^{0}|P=1 \right]\cdot E\left[ P \right]}{1-E\left[ P \right]}\geq1$, we have

$$CSR \leq\frac{1-E\left[ Y^{1}|P=0 \right]}{1-E\left[ Y^{0}|P=0 \right]}\overset{\left( D4 \right)}{=}{CSR}_{P=0}.$$

**Simulation**

We compared the marginal effects with the effects among the non-prevalent cases. To do so, we arbitrarily assigned risks for the outcome under treatment and control among individuals who do not have the condition of interest at baseline. Specifically, we considered all possible pairs of risks in the set 0.01, 0.05, 0.1, 0.3, 0.5. To facilitate visualization, we only considered settings in which the treatment reduces the risk of the outcome, that is, when the risk of the outcome under treatment is lower than the risk of the outcome under control among non-prevalent cases.

We studied different proportions of prevalent cases at baseline, ranging from 0 to 0.9. Prevalent cases were assumed to have a risk of 1 of experiencing the outcome under both treatments (which ensures that Assumption 3 holds). For each scenario, we then calculated the marginal risk of the outcome under treatment and under control, which is a weighted average of the corresponding risks in the subgroups of prevalent cases and non-prevalent cases. Finally, we calculated different causal effects in the entire population and in the population that excludes the prevalent cases. The results are visualized in Figure S1.

As expected from our theoretical results, the causal survival ratio remained the same when excluding prevalent cases, regardless of the proportion of prevalent cases. Even when prevalent cases represented 90% of the entire population, the survival ratio remained unchanged. The causal risk ratio, however, changed dramatically when excluding prevalent cases; the discrepancy increased with the proportion of prevalent cases in the population. In the entire population, the causal risk difference and the causal risk ratio approached 0 and 1, respectively (suggesting no effect), as the proportion of prevalent cases increased. However, when the marginal risk of the outcome under control was low, the marginal causal risk difference was similar to the one calculated excluding prevalent cases. As expected from Proposition 1.

In Figure S2, we repeated the calculations in a setting where Assumption 3 was violated. Here, we assigned a risk of 1 to prevalent cases under treatment (as in Assumption 4), but only 0.80 under control. In this simulation, the causal survival ratio was not stable to the exclusion of prevalent cases. When the proportion of prevalent cases was very low and the risk under control was low, the risk difference was approximately stable. However, in this new simulation, there was a larger difference between marginal effects and effects calculated excluding the prevalent cases, especially when the proportion of prevalent cases was high. As expected from Proposition 2, the marginal survival ratio was always lower than or equal to the one among non-prevalent cases only.

**Supplementary figures**


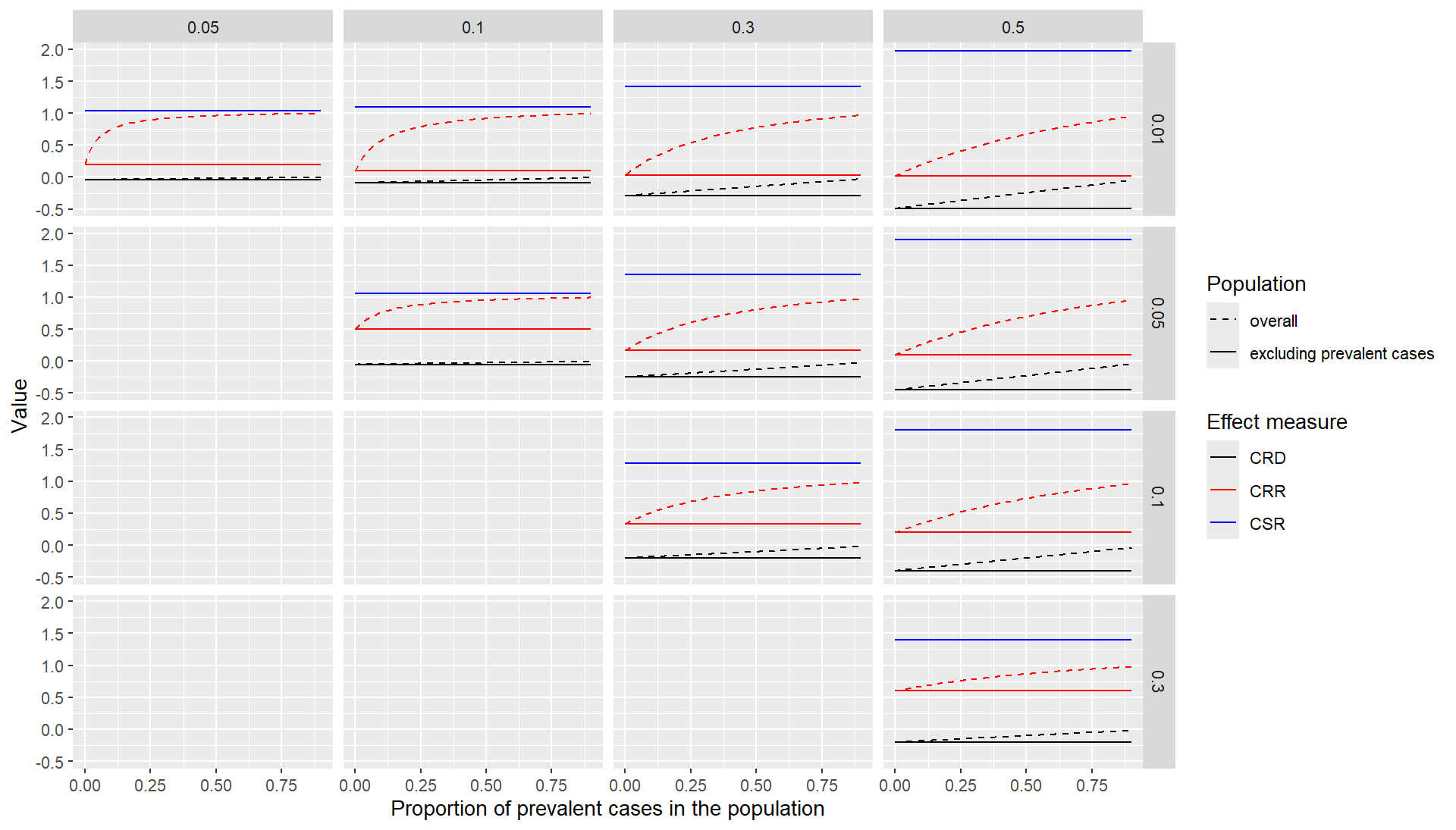

**Figure S1.** Results of the simulation. Causal risk difference (in black), Causal risk ratio (in red), Causal survival ratio (in blue) calculated both marginally (dashed line) and only among non-prevalent cases (continuous line). Different scenarios are considered. The scenarios are defined by: the proportion of prevalent cases in the population, $E\left[ P \right]$ (x-axis;); the risk of the outcome under control among non-prevalent cases, $E\left[ Y^{0}|P=0 \right]$ (columns); and the risk of the outcome under treatment among non-prevalent cases, $E\left[ Y^{1}|P=0 \right]$ (rows). We considered only scenarios in which the treatment reduces the risk, this is why no effects are reported for certain combinations. Prevalent cases are assumed to have a risk of one under both treatments ($E\left[ Y^{1}|P=1 \right]=E\left[ Y^{0}|P=1 \right]=1$).


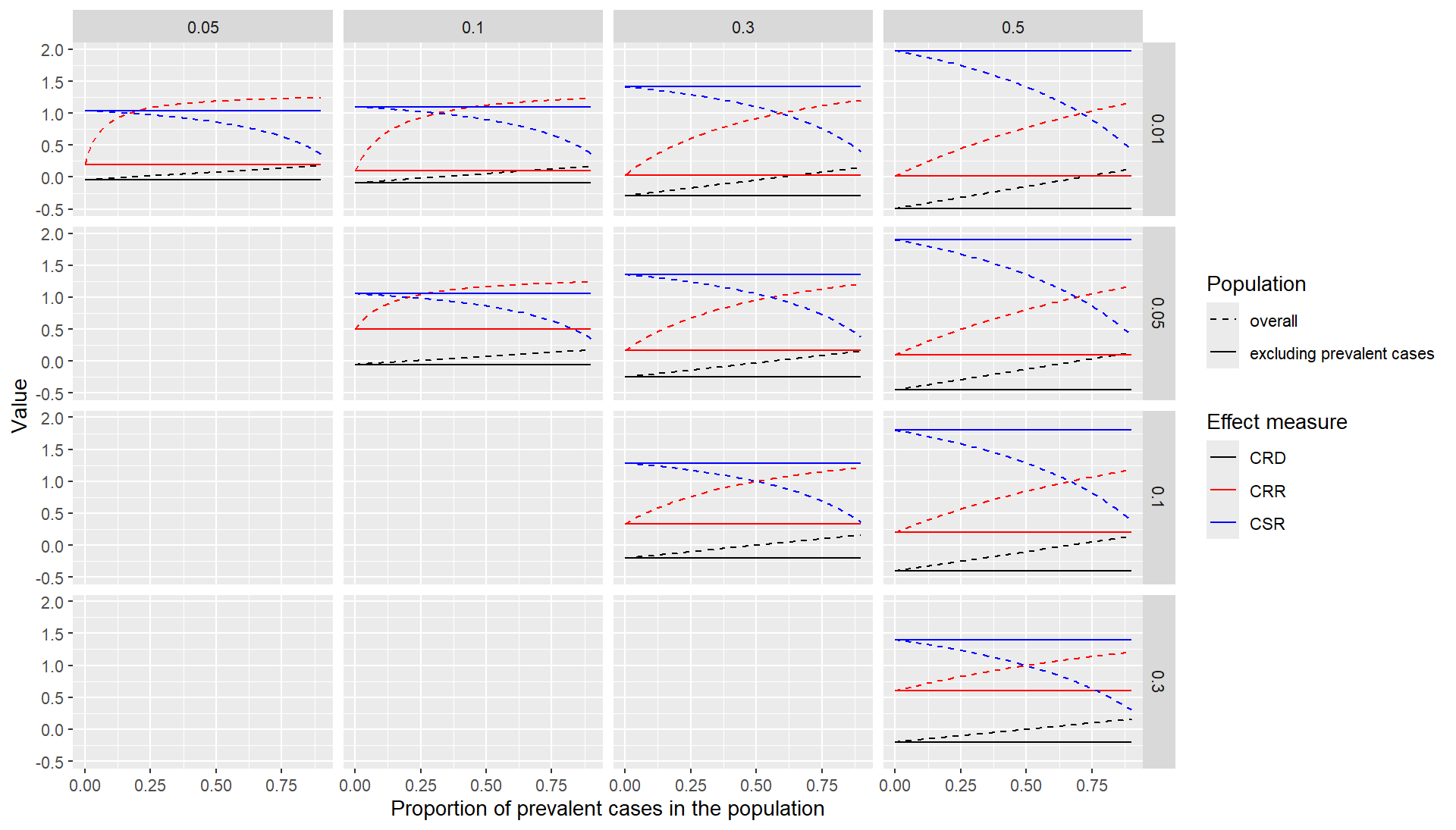

**Figure S2.** Results from the same calculations for Figure S1, but relaxing the assumption that prevalent cases always experience the outcome under both treatments. In this simulation, the risk of the outcome for prevalent cases was 1 under treatment ($E\left[ Y^{1}|P=1 \right]=1$), and 0.8 under control ($E\left[ Y^{0}|P=1 \right]=0.8$).

**References**

1. Hernán MA, Robins JM. Causal Inference: What if. Boca Raton: Chapman & Hall/CRC; 2020.

2. Huitfeldt A, Fox MP, Murray EJ, Hróbjartsson A, Daniel RM. Shall we count the living or the dead? [Internet]. arXiv [stat.ME]. 2021. Available from: http://arxiv.org/abs/2106.06316

3. Brenner H, Heisser T, Cardoso R, Hoffmeister M. When gold standards are not so golden: prevalence bias in randomized trials on endoscopic colorectal cancer screening. Eur J Epidemiol. 2023;38:933–7.

4. Bretthauer M, Løberg M, Wieszczy P, Kalager M, Emilsson L, Garborg K, et al. Effect of colonoscopy screening on risks of colorectal cancer and related death. N Engl J Med. 2022;387:1547–56.

5. Brenner H, Heisser T, Cardoso R, Hoffmeister M. The underestimated preventive effects of flexible sigmoidoscopy screening: re-analysis and meta-analysis of randomized trials. Eur J Epidemiol. 2024;39:743–51.

6. Piccininni M, Stensrud MJ. Immune-selection stability is a neglected property of the causal risk ratio. Am J Epidemiol [Internet]. 2025; Available from: http://dx.doi.org/10.1093/aje/kwaf086
